# Extracellular Vesicles for the Diagnosis and Treatment of Parkinson’s Disease

**DOI:** 10.14336/AD.2021.0516

**Published:** 2021-09-01

**Authors:** Raghavendra Upadhya, Ashok K Shetty

**Affiliations:** Institute for Regenerative Medicine, Department of Molecular and Cellular Medicine, Texas A&M University College of Medicine, College Station, Texas, USA

**Keywords:** alpha-synuclein, dopamine, dopaminergic neurons, exosomes, extracellular vesicles, EV engineering, EV targeting, motor deficits, neuroinflammation, Parkinson’s disease, substantia nigra

## Abstract

Extracellular vesicles (EVs) shed by neurons and glia in the central nervous system carry a cargo of specific bioactive molecules, facilitating intercellular communication. However, in neurodegenerative disease conditions, EVs carry pathological miRNAs and/or proteins involved in spreading the disease. Such EVs are also found in the cerebrospinal fluid (CSF) or the circulating blood, the characterization of which could identify biomarkers linked to specific neurodegenerative diseases. Moreover, EVs secreted by various stem/progenitor cells carry therapeutic miRNAs and proteins, which have shown promise to alleviate symptoms and slow down the progression of neurodegenerative diseases. The ability of exogenously administered EVs to easily cross the blood-brain barrier with no risk for thrombosis and incorporate into neurons and glia has also opened up the possibility of using nano-sized EVs as carriers of therapeutic drugs or bioactive proteins. This review summarizes the role and function of EVs in alpha-synuclein-mediated neurodegeneration and the spread of alpha-synuclein from neurons to glia, leading to the activation of the inflammatory response in Parkinson’s disease (PD). Moreover, the promise of brain-derived EVs in the CSF and the circulating blood for biomarker discovery and the efficacy of stem/progenitor cell-derived EVs or EVs loaded with bioactive molecules such as dopamine, catalase, curcumin, and siRNAs, in alleviating Parkinsonian symptoms are discussed.

## 1. Introduction

Parkinson's disease (PD), an advancing movement disorder and the most common neurodegenerative disease after Alzheimer’s disease, afflicts ~2% of the world’s population aged >65 years [1]. PD symptoms comprise a wide variety of motor symptoms, including resting tremor, bradykinesia, muscular rigidity, and postural instability. The neuropathological changes include a progressive loss of dopaminergic neurons in the substantia nigra pars compacta and the intracytoplasmic aggregation of the synaptic protein alpha-synuclein (α-syn), termed Lewy bodies (LBs) and Lewy neurites (LNs) in surviving neurons [2]. However, other brain regions, including the olfactory bulb, neocortex, limbic system, and brainstem nuclei, have also been implicated in PD pathogenesis, suggesting that PD displays complex pathology [3]. These changes reduce the concentration of neurotransmitter dopamine (DA) in the nigrostriatal pathway, cause an imbalance between acetylcholine (ACH) and dopamine (DA) and lead to motor problems [1, 4, 5]. In addition to the fibrillary aggregation of α-syn, modifications such as elevated reactive oxygen species (ROS), chronic neuroinflammation, excitotoxicity, and loss of critical neurotrophic factors in the brain promote both genetic and sporadic forms of PD [6]. Studies have also revealed the trans-neuronal transmission of aggregated α-syn, as the pathogenic forms of α-syn secreted into the extracellular space spreads to neurons and non-neuronal cells in neighboring regions [3]. Nevertheless, the particular mechanisms by which the secreted α-syn is transmitted to neural cells at proximal and distant sites remain to be elucidated. A series of studies have recently indicated the role of extracellular vesicles (EVs) in the transmission of α-syn, both within the nigrostriatal system and the other brain regions [7-9].

The EVs, a heterogeneous group of nano-sized membranous structures of biological origin [10], serve a vital role in cell-to-cell communication [11]. Although initially thought of as a vehicle by which cells get rid of unneeded materials generated during the metabolic processes [12], EVs received considerable attention once their cargo containing nucleic acids, proteins, lipids, and small molecules, and their ability to influence physiological states of cells in distant places from the site of origin were discovered [13-15]. The role of EVs in the central nervous system (CNS) has been demonstrated in processes such as neurodevelopment, synaptic communication, myelin formation, neurite growth, and neuronal survival [16-22], which highlighted the significance of EVs in tissue repair and regeneration.

Additional studies have also demonstrated the complex roles played by EVs in the CNS, including the propagation of pathogenic proteins, such as amyloid-beta (Aβ) and p-tau in AD, and α-syn in PD [23], contributing to exacerbation of the disease pathophysiology. EVs have also been implicated in spreading disease-causing miRNAs and mRNAs [24]. For example, EVs from diseased patients carry elevated levels of specific miRNAs, which may be involved in causing neuroinflammation or neurodegeneration, leading to cognitive, memory, and mood dysfunction [25-27]. Hence, EVs play crucial roles in genetic information transfer in both healthy and disease states. On the other hand, EVs secreted by stem/progenitor cells carry therapeutic miRNAs and proteins, which could provide neuroprotection, reduce oxidative stress and neuroinflammation, increase neurogenesis, and improve cognitive and mood function in disease states [19-21, 28-32]. From this perspective, currently, there is a considerable interest to use EVs secreted from a variety of stem/progenitor cells and EVs fortified with unique miRNAs, mRNAs, and proteins to stimulate tissue repair and regeneration after injury or disease. Some studies have also utilized EVs’ natural ability as vehicles to deliver therapeutic materials into target cells in various organs. This review deliberates the role of brain-derived EVs in PD progression and the utility of brain-derived and other tissue-derived EVs in body fluids for diagnosis or ascertaining the PD progression. The last section discusses the prospects of stem cell-derived EVs for treating PD.

## 2. Role of EVs in the pathogenesis of PD

All neural cells, including neurons, astrocytes, microglia, and oligodendrocytes, can shed EVs, which likely influences the recipient cells' physiological functions by transferring of proteins, lipids, and nucleic acids [33]. Therefore, it is presumed that EVs secreted by cells in pathological states carry and spread the misfolded, toxic, pathological molecules [34]. Indeed, misfolded toxic proteins' propagation has been validated in PD through both *in vitro* and *in vivo* experiments [7, 35]. Of particular interest is the α-syn propagation along several brain regions, which is believed to be one of the central mechanisms underlying PD progression [36-39]. Lee and colleagues reported a vesicle-mediated release of both monomeric and oligomeric forms of α-syn in response to faulty proteasomal and mitochondrial dysfunction seen in the pathogenesis of PD [40]. Additional studies found an increased release of EVs containing α-syn when intracellular protein trafficking through lysosomes was blocked, confirming that lysosomal dysfunction is likely one of the major factors accelerating PD pathology [41-43]. Inhibition of glucocerebrosidase 1 (GBA-1) activity has also increased the release of brain EVs containing α-syn oligomers [44]. Furthermore, vesicular α-syn is more prone to aggregation than cytosolic α-syn, indicating disease spread in a prion-like fashion from cell to cell. A study by Grey and associates further suggested that the presence of high gangliosides (gangliosides, GM1 and GM3) within EVs are making α-syn more prone to aggregation [45].

A study using cultured SH-SY5Y neuroblastoma cells demonstrated the release of both monomeric and oligomeric forms of α-syn through EVs [7]. Notably, the recipient cells incorporated EVs containing α-syn more efficiently than soluble α-syn in the conditioned media, which resulted in neurodegeneration [42]. EVs’ role has also been seen in α-syn transfer from SH-SY5Y cells overexpressing vesicular α-syn to normal SH-SY5Y cells, which supported the concept of α-syn from disease-affected neurons getting transported to healthy unaffected neurons in other regions of the nervous system [41]. Such spreading of α-syn could also be gleaned from the results in a grafting paradigm where α-syn from the host mouse neurons displaying PD pathology were transmitted to graft-derived dopaminergic neurons [46]. There is still some ambiguity regarding the amount of EV-bound α-syn because it varied between different synucleinopathies, with some studies showing increased and other studies reporting decreased EV-bound α-syn in PD patients [35, 47, 48]. Nonetheless, EV-bound α-syn has been shown to induce oligomerization, which was evident from the ability of α-syn in EVs isolated from the cerebrospinal fluid (CSF) to promote oligomerization in a dose-dependent manner, and a strong correlation between the amount of EVs with α-syn in the CSF and the severity of the cognitive impairment in PD patients [35].

A recent study probed microglia's contribution to α-syn aggregation and spread [8]. When challenged with human α-syn preformed fibrils, microglia released EVs containing α-syn, which induced α-syn aggregation in the recipient neurons. Furthermore, in the presence of proinflammatory cytokines such as TNF-α, IL-1β, or IL-6, such EVs amplified α-syn aggregation in neurons. However, a reduced α-syn transmission was observed when EV synthesis was inhibited in microglia. Moreover, direct injection of EVs shed from microglia treated with α-syn preformed fibrils into the mouse striatum resulted in phosphorylated α-syn aggregation in several brain regions connected with the striatum, degeneration of dopaminergic neurons, and motor deficits [8]. Interestingly, such α-syn transmission and neurodegeneration could be suppressed through microglia depletion. The study also found that microglia-derived EVs in the CSF of PD patients contained α-syn oligomers capable of inducing α-syn aggregation in neurons. Overall, the study suggested that microglia-derived EVs are involved in the progression of α-syn pathology in PD, and microglia-derived EVs carrying α-syn oligomer are adequate to induce motor deficits and neuropathology of sporadic PD [8].

In addition to the propagation of α-syn, EVs have also been implicated in the transfer of genes carrying causal mutations or variants acting as risk factors for developing PD. These include the transfer of mutated leucine-rich repeat kinase 2 (LRRK2) linked to late-onset PD, which can interact with Rab proteins, co-localize with multi-vesicular bodies (MVBs) [49], and get released into the extracellular milieu through EVs [50-52]. A study has also shown that a mutation (R1441C LRRK2) resulted in the formation of abnormally larger MVBs [49], facilitating the release of a large amount of toxic form of α-syn through EVs and causing an increased spread of the disease. Moreover, the tau gene (MAPT), involved in AD's pathogenesis, can also increase the genetic risks for PD [52, 53]. Giasson and colleagues showed that a variant of tau protein could interact with α-syn and promote its oligomerization and toxicity [54]. Another protein linked to EV biology and PD pathogenesis is the mutated vacuolar protein sorting-associated protein 35 (VPS35), implicated in the late-onset autosomal dominant PD. VPS35 is also an essential component of the retrograde transport system involved in transporting materials from the endosome to the Golgi apparatus. Since such a defective retrograde system could increase EV secretion [55], it has been speculated that VPS35-driven increased EV release likely contributes to increased extracellular α-syn levels. Interactions of α-syn with small-ubiquitin-like modifier (SUMO) has also been suggested to regulate α-syn recruitment to EVs [56, 57].

In summary, the studies discussed above support the role of EV-bound α-syn in the spread of α-syn to healthy neighboring cells, which likely promotes further α-syn aggregation, causing neurodegeneration. However, in PD, many non-catecholaminergic neurons in close anatomical proximity of degenerating Lewy body-containing neurons are spared. It is plausible that the Lewy body-containing dopaminergic neurons release EVs expressing specific surface ligands to incorporate only into certain types of neurons or glia resulting in the sparing of non-catecholaminergic neurons. However, such a possibility remains to be confirmed. Thus, EVs containing α-syn released by injured neurons could be transmitted from neuron-to-neuron, leading to α-syn spread, and from neuron-to-glia leading to the activation of an inflammatory response. Likewise, EVs released by activated glial cells containing inflammatory mediators can be transmitted from glia-to-glia leading to the propagation and perpetuation of the inflammatory response. Such processes could exacerbate neuroinflammation and contribute to neuronal dysfunction, neurodegeneration, and disease progression [58].

## 3. EVs in body fluids as biomarkers of PD

**Figure 1.**
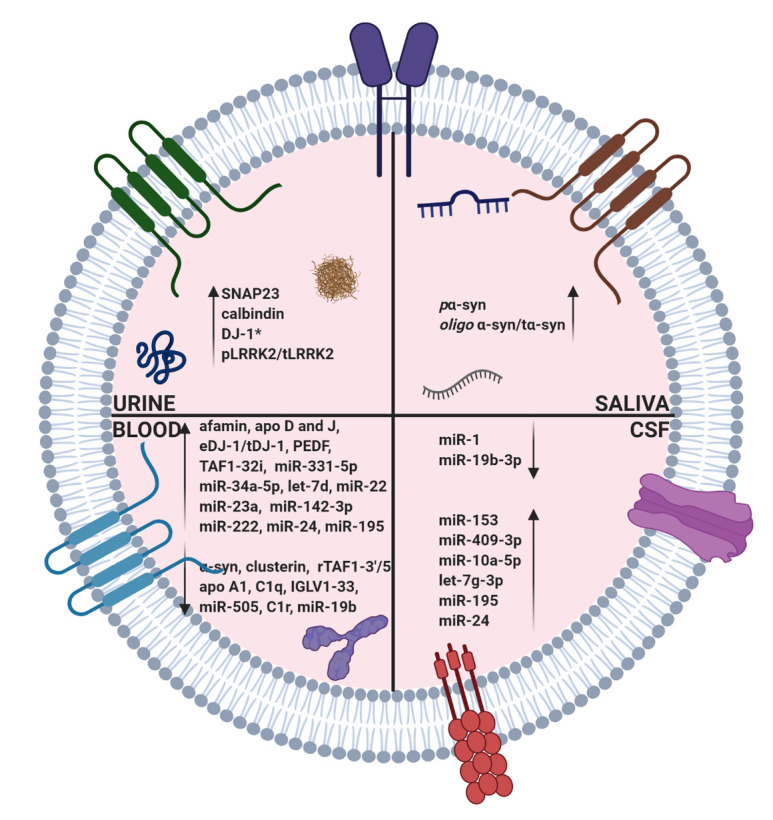
***A schematic showing an EV as an alternative source of biomarkers in Parkinson’s disease (PD).* Brain-derived extracellular vesicles (EVs) in the blood of PD patients have displayed downregulation of afamin, Apo D ratio of eDJ-1/tDJ-1, PEDF, miR-331-5p, let-7d, miR-22, miR-23a, miR-24, miR-142-3p, miR-222, and upregulation of alpha-synuclein, clusterin, apo A1, C1q, IGLV-1-33, miR-505, and C1r**. EVs from the cerebrospinal fluid of PD patients exhibited downregulation of miR-1, miR-19b-3p, miR-19b, miR-153, miR-409-3p, miR-10a, let-7g-3p, miR-195, and miR-24. Furthermore, in EVs collected from the urine of PD patients, upregulation of SNAP23, calbindin, DJ-1, and ratio of pLRRK2/tLRRK2 have been reported. Additionally, EVs from saliva displayed upregulation of phosphorylated α-syn and a higher ratio of oligomeric α-syn to total α syn. EV lipids, on the other hand, have not been explored in the diagnosis or prognosis of PD. PD, Parkinson’s disease; pLRRK2, phosphorylated LRRK2; tLRRK2, total LRRK2; eDJ-1, exosomal DJ-1; tDJ-1, total DJ-1; oligo α-syn, oligomeric α-syn; DJ-1*, DJ-1 amount in male PD patients.

Based on clinical signs and rating of motor functions, the current PD diagnosis is efficient only after ~70% loss of DA-ergic neurons in the substantia nigra [59]. Detection of the earliest phases of PD will require reliable prognostic markers. In this regard, EVs carrying the toxic misfolded or mutated proteins and miRNAs could be useful as potential biomarkers of PD. Characterization of disease-related constituents of EVs isolated from the urine, blood, saliva, or CSF of PD patients has suggested that EVs’ analysis could predict the course of PD progression and help in the application of disease-modifying treatments [16]. A schematic showing an EV found in the CSF, blood, saliva, or urine of PD patients as an alternative source of biomarkers in PD is illustrated in Fig. 1.

### 3.1. CNS-derived EVs in the CSF of PD patients

The CSF is an ideal source for PD diagnostic markers because of its proximity to the CNS, although CSF collection can be invasive compared to other body fluids. The previous investigations measuring α-syn concentration directly from the CSF were not successful in distinguishing PD patients from healthy volunteers [60-63]. However, Stuendl and associates demonstrated the value of characterizing EVs isolated from the CSF of patients in different PD stages and proposed that EV-bound α-syn could be advantageous as a potential biomarker [35, 57]. Additional in vitro studies demonstrated that α-syn found in CSF-derived EVs induced oligomerization of soluble α-syn in cells taking up EVs, implying that EV-bound α-syn can act as a seed. Thus, α-syn analysis in CSF-derived EVs serves as a useful biomarker for α-syn-related neurodegenerative disorders [35]. Furthermore, the various RNA species present in EVs would likely provide further insights into disease mechanisms because miRNAs within CSF-derived EVs were more stable and reliable than free miRNAs in the CSF [64].

A study evaluating the presence of miRNAs in EVs from the CSF of PD patients reported upregulation of 16 miRNAs and downregulation of 11 miRNAs compared to healthy controls [65]. The levels of miRNA-153, miRNA-409-3p, miRNA-10a- 5p and let-7g-3p were elevated whereas miRNA-1 and miRNA-19b-3p were downregulated. These miRNAs target genes involved in crucial pathways implicated in PD, such as neurotrophin signaling and dopaminergic synapses [65]. Another study identified 24 miRNAs dysregulated in the serum and CSF of PD patients. They found that miRNA- 19b was downregulated and miRNA-195 and miRNA-24 were upregulated in PD patients [66]. Overall, analysis of EVs from the CSF generally seemed useful for identifying biomarkers related to PD. However, it was unclear whether specific stages of PD could be diagnosed through analyses of the miRNA and/or protein cargo of EVs found in the CSF. Also, due to the requirement of an invasive procedure to collect CSF, attention has turned to identify the biomarkers in blood-derived EVs.

### 3.2. Analysis of blood-derived EVs in PD patients

Initially, since α-syn is also carried by blood cell-derived EVs [47], the characterization of α-syn in blood-derived EVs did not lead to biomarker discovery. However, Shi and colleagues purified CNS-specific EVs in the blood by selectively precipitating L1 cell adhesion molecule (L1CAM) positive EVs and demonstrated that α-syn in such EVs are derived from the CNS [48]. They also showed that the concentration of α-syn in CNS-derived EVs in the plasma is substantially higher in PD patients and α-syn levels in such EVs correlated with the disease severity, which supported the value of characterizing α-syn in CNS-derived EVs from the plasma as a biomarker of PD [48]. Another study reported significant downregulation of clusterin, apolipoprotein A1, and complement C1r subcomponent in EVs from the plasma of PD patients at Hoehn and Yahr (HY) stages II and III compared to healthy controls [67], suggesting that these proteins may also be used as candidate biomarkers for PD. Moreover, a study by Jiang and associates showed increased expression of afamin, apolipoprotein D and J, and pigmented epithelium-derived factor, and reduced expression of complement C1q and immunoglobulin lambda variable 1-33 (IGLV1-33) in blood-derived EVs from PD patients [68]. The other significant markers identified in CNS-derived EVs in PD patients' blood include DJ-1 and LRRK2 linked to familial and sporadic PD [69]. Additional studies on blood-derived EVs in PD patients have reported higher miR-331-5p and lower miR-505 [70], and upregulation of let-7d, miR-22, miR-23a, miR-24, miR-142-3p, miR-222.

A study revealed higher levels of miR-34a-5p in small EVs derived from the plasma of PD patients, and receiver operating characteristic curve (ROC curve) analysis implied an excellent diagnostic value in differentiating PD patients from controls [71]. Also, miR-34a-5p levels in EVs positively correlated with PD duration, Hoehn and Yahr, and Beck Depression Inventory scores. A study surveying mitochondrial signatures such as ATP5A, MTCOI, NADH, NDUFB8, NDUFS3, SDHB, UQCRC2 in blood-derived small EVs from PD and non-PD controls advocated that EVs may provide potential biomarkers in PD diagnosis and aid identification of potential targets for personalized medicine [72]. Another study found no differences in neurofilament light-chain (NfL) content in plasma-derived EVs between PD patients and healthy controls, but there was a trend of increased NfL in PD patients with severe akinetic rigidity. Such finding suggested that NfL content in plasma-derived EVs could serve as a biomarker of motor symptom severity [73]. Vacchi and colleagues, through analysis of surface markers related to immune and inflammatory cells in plasma-derived EVs, reported that EVs from PD patients displayed overexpression of immune markers and suggested that such characterization could provide biomarkers for an optimal diagnosis of PD [74].

Thus, analyses of both CNS-derived and other cell-derived EVs in PD patients' blood could provide insights on specific biomarkers and the progression of the disease. Nonetheless, longitudinal studies are yet to be done to validate the potential biomarkers that could be utilized to diagnose PD or to assess the severity of PD pathogenesis and how that correlates with motor and non-motor symptoms. Also, there are limitations to using CNS-derived EVs in the blood as a diagnostic tool. The currently available methods for quantification of alpha-synuclein, p-alpha-synuclein, or other products in brain-derived EVs in the blood are not optimal for distinguishing PD from other movement disorders such as the essential tremor, neurodegeneration with brain iron accumulation, dystonic tremor, or multiple system atrophy.

### 3.3. EVs in the saliva and urine of PD patients

The EVs derived from PD patients' saliva have also been shown to help to identify biomarkers, especially EVs carrying α-syn. It has been reported that salivary glands shed EVs containing α-syn into the saliva [47], and EVs derived from PD patients' saliva displayed a higher level of α-syn_Olig_, a higher ratio of α-syn_Olig_/α-syn_Total_ [75], and a higher concentration of phosphorylated α-syn [76], compared to EVs in the saliva of healthy controls. However, in these studies, the amount of α-syn_Olig_, α-syn_Olig_/α-syn_Total_ in salivary EVs did not correlate with features such patients’ age, the age of PD onset, and the disease duration. Longitudinal investigatons are required in the future to determine whether α-syn levels in salivary EVs increase with PD progression and severity. Furthermore, studies characterizing EVs from the urine suggested upregulation of synaptosomal-associated protein 23 (SNAP23) and calbindin along with striking enrichment of endolysosomal proteins in PD patients [77], suggesting the utility of EVs in the urine as an accessible source of biomarker discovery for PD. Another study reported DJ-1 and LRRK2 but not α-syn in urine-derived EVs from PD patients [78]. The study found a ~1.7-fold increase in the DJ-1 protein in male PD patients compared to healthy male counterparts [78]. Fraser and colleagues found an increased ratio of Ser(P)-1292 LRRK2 to total LRRK2 in urine-derived EVs from 79 idiopathic PD patients compared to 79 healthy controls [79]. The study also revealed that Ser(P)-1292 LRRK2 levels correlated with the severity of cognitive impairments. These findings have considerable significance because a mutation in LRRK2 enhances the level of autophosphorylated LRRK2 (Ser(P)-1292 LRRK2) protein, which is the most common cause of inherited PD. Thus, analysis of (Ser(P)-1292 LRRK2 in EVs could serve as a helpful biomarker for both familial and idiopathic PD.

In summary, a careful analysis of EVs in the CSF, blood, saliva, and urine of PD patients seems to aid PD diagnosis. However, it remains to be determined whether the concentration of potential biomarkers found in salivary or urine EVs correlate with the extent of similar biomarkers in brain-derived EVs in the CSF or the blood. Moreover, characterization of EVs derived from specific brain cells (e.g., dopaminergic neurons, reactive astrocytes, or activated microglia) in the blood might provide an even higher resolution for diagnosis and biomarker discovery in PD. In addition, analyses of samples from a large number of patients are needed to verify the specificity and sensitivity of various biomarkers. Cutting-edge techniques that isolate and characterize specific types of EVs (e.g., EVs shed by dopaminergic neurons) without contaminants also need to be developed.

## 4. Promise of stem cell-derived EVs for treating PD

Novel therapeutic approaches capable of restraining the disease progression and improving brain function have immense value for improving the condition of PD patients. As in many other brain disorders, lack of adequate penetrance of the blood-brain barrier (BBB) has been an issue for delivering PD therapeutics into the brain. EVs offer an attractive platform for delivering proteins, mRNAs, and miRNAs or drugs to the CNS due to their natural ability to transport biomolecules intercellularly, protect their content, and cross the brain capillary endothelial cells or the BBB [80]. From these perspectives, EVs derived from the stem or progenitor cells have been explored to repair or rejuvenate the brain either through effects mediated by their natural cargo of miRNAs and proteins or engineered cargo. Indeed, many studies have reported beneficial effects of administering the stem/progenitor cell-derived EVs in a variety of neurological and neurodegenerative disease models, with specific therapeutic outcomes depending on the properties of parental cells or culture conditions in which the EVs were derived [19-21, 28, 29]. Here, we discuss prominent findings from studies that tested the efficacy of EVs in PD models and the promise and challenges in developing EV-based therapy for PD.

A study by Jarmalaviciute and colleagues examined EVs' neuroprotective properties in an in vitro system involving 6-hydroxy-dopamine (6-OHDA) induced toxicity on dopaminergic neurons. In this study, the EVs were isolated from cultures of dental pulp mesenchymal stem cells (MSCs) from the human exfoliated deciduous teeth. The addition of EVs to cultures protected neural stem cell-derived dopaminergic neurons against 6-OHDA [81]. A follow-up study demonstrated that intranasally administered MSC-EVs improved therapeutic outcome in a 6-OHDA model of rat PD, where 6-OHDA was directly injected into the medial forebrain bundle (MFB). Specifically, 15 days of EV treatment (once daily for a total of 5.13 x 10^9^ billion) commencing eight days after a unilateral administration of 6-OHDA into MFB significantly improved several gait parameters and improvements in the overall motor function [82]. Also, improvements in the gait parameters correlated with the normalization of tyrosine hydroxylase (TH)-positive fibers in the striatum and the substantia nigra. Thus, the study provided evidence that EVs from MSCs could reduce parkinsonian symptoms in a rat prototype [82]. In another study, the administration of conditioned media from the cultures of human dental pulp mesenchymal stem cells (MSCs) in a PD model also resulted in similar effects [83].

Chen and colleagues first investigated the efficacy of EVs generated from human umbilical cord MSCs (hUCMSCs) to prevent apoptosis in cultures of the human neuroblastoma cell line (SH-SY5Y) against the 6-OHDA challenge [84]. In this study, hUCMSCs with SH-SY5Y cells were co-cultured in a transwell system that prevented the direct cell to cell contact but allowed the exchange of secreted vesicles. 6-OHDA treatment to SH-SY5Y cells in such a system prevented apoptosis and stimulated the proliferation of SH-SY5Y cells. However, when cultures were treated with an exosome release inhibitor (GW4869), the beneficial effects were attenuated, suggesting that the protection and proliferation of SH-SY5Y cells are mediated by EVs secreted by hUCMSCs [84]. Next, the effect of intravenous administration of hUCMSC-EVs (200 µg, every 3^rd^ day for eight weeks) was examined in a 6-OHDA rat model of PD. EVs crossed the BBB and got incorporated into the substantia nigra, which reduced apoptosis, significant protection of dopaminergic neurons, and increased dopamine concentration in the striatum. The EV-treated animals also displayed diminished asymmetric rotation in response to apomorphine treatment at eight weeks after transplantation [84]. However, the study did not characterize the miRNA/protein cargo of EVs employed in this study, and hence the types of miRNAs or proteins that mediated neuroprotection in this study are unknown.

## 5. Potential of Engineered EVs for treating PD

As inherently shed EVs carry a cargo containing proteins and miRNAs from parental cells to recipient cells efficiently, EVs are considered attractive vehicles for delivering therapeutic proteins and miRNAs. EV-based drug delivery has several advantages, including reduced immune response if derived from the same species, protection of the cargo from degradation and stability in biologic fluids, and the ability to incorporate into target cells. Therapeutic EVs such as those derived from MSCs and neural stem cells (NSCs) could also be modified using molecular engineering techniques to carry protein ligands for targeted delivery to specific cells in the body. EVs can also be loaded with exogenous drugs. Endogenously, the cargo of EVs can be modified by overexpressing or knocking down specific mRNAs, miRNAs, or proteins in the parental cells to exploit the cellular machinery for cargo sorting into EVs. Such techniques facilitate the production of EVs carrying specific therapeutic proteins, siRNAs for selectively knocking toxic proteins, or carrying mRNAs encoding neurogenic growth factors [85]. Studies that have examined the efficacy of such approaches in PD models are discussed in the next section.

### 5.1. Efficacy of EVs overexpressing a specific protein in a PD model

While Lewy bodies containing α-syn is the classical hallmark of PD pathogenesis, other significant pathological changes likely also contribute to progressive neurodegeneration and motor dysfunction in PD. These include sustained chronic neuroinflammation with activated microglia cells and increased release of the pro-inflammatory cytokines, and elevated concentration of reactive oxygen species (ROS) associated with reduced antioxidants such as catalase, glutathione, and superoxide dismutase. Haney and associates loaded a mouse macrophage cell line (Raw 264.7)-derived EVs with the ROS scavenger protein catalase [86]. In a cell culture system, macrophages stimulated with lipopolysaccharide (LPS), and tumor necrosis factor-α (TNF-α) displayed reduced ROS when cultures were treated with catalase-loaded EVs. Administration of catalase-loaded EVs in a 6-OHDA mouse model of PD reduced activated microglia compared to free catalase administration, suggesting that EV-based delivery of catalase has promise for restraining oxidative stress and neuroinflammation in PD [86].

### 5.2. Effects of EVs overexpressing specific mRNA/siRNA/shRNA or loaded with drugs in PD models

Cooper and colleagues examined the efficacy of targeted delivery of siRNA in a model of PD using EVs expressing rabies virus glycoprotein (RVG) [87]. Murine dendritic cells derived from the bone marrow were first transfected with RVG-lysosome-associated membrane protein 2 (Lamp-2) plasmids to make EVs expressing RVG. Then, siRNAs designed to target α-syn were loaded onto EVs using electroporation. Intravenous administration of siRNA-loaded EVs in mice expressing human phosphorylation-mimic S129D α-syn resulted in a significant reduction in both α-syn mRNA and protein throughout the brain at seven days after the administration. Such intervention also reduced intraneuronal α-syn protein aggregates, particularly in dopaminergic neurons [87]. A follow-up in vitro study demonstrated the ability of shRNA-loaded EVs expressing RVG to downregulate α-syn in SH-SY5Y cells with overexpression of S129D α-syn [88]. Furthermore, intravenous administration of such EVs reduced both α-syn mRNA and protein levels in transgenic mice expressing the phosphomimetic human S129D alpha-synuclein, reduced the loss of dopaminergic neurons, and alleviated PD symptoms, implying the therapeutic benefits of such EVs to treat PD [88]. Kojima and coworkers engineered EVs with catalase mRNA and an element that coded RVG peptide for the selective targeting of brain cells and L7Ae for mRNA packaging. Such genetic modification of parental cells resulted in EVs with catalase mRNA and RVG-Lamp-2 expression, which facilitated catalase mRNA delivery into the cytosol of target cells. Delivery of catalase mRNA reduced neurotoxicity and neuroinflammation in both *in vitro* and *in vivo* models of PD, indicating the potential usefulness of such EVs for RNA delivery-based therapeutic applications [89]. Qu and associates used EVs isolated from the blood to load and deliver dopamine into the brain in a PD model. Dopamine-loaded EVs crossed the BBB and delivered DA to the striatum and substantia nigra. Administration of dopamine-loaded EVs resulted in over 15 folds higher dopamine distribution in the brain, a higher therapeutic efficacy, and lower systemic toxicity than the administration of free dopamine, suggesting that EV-based drug delivery could be an efficient treatment option in the management of PD [90].

In another study, Liu and co-workers engineered a nano-scavenger called “REXO-C/ANP/S” for delivering drugs through the BBB into neurons [91]. The system comprised a shell called REXO and a reactive oxygen species (ROS)-responsive gene-chem coloaded core or nanocomplex. REXO is a targeted immature dendritic cell-derived exosome with a modified RVG peptide to facilitate its binding to acetylcholine receptors on neurons and the BBB. The nanocomplex comprised an amphiphilic polymer encapsulating the hydrophobic drug curcumin and siRNA targeting *SNCA* gene involved in alpha-synuclein production. Such an engineered delivery system could cross the BBB and target and release drugs directly to dopaminergic neurons in a high ROS milieu [91]. When administered in a mouse model of PD, REXO-C/ANP/S targeted TH+ dopaminergic neurons in the substantia nigra and enhanced α-syn clearance, which resulted in reduced motor deficits. Additional analysis validated the role of the curcumin in quenching ROS production and dampening neuroinflammation. Furthermore, REXO-C/ANP/S significantly increased TGF-β and IL-10 levels, resulting in antiinflammatory and neuroprotective effects [91]. Thus, EVs engineered to pass through the BBB and carry specific therapeutic molecules directly to neurons have promise for treating PD and other neurodegenerative disorders.

## 6. Conclusions and future perspectives

In PD, EV-mediated release of both monomeric and oligomeric forms of α-syn, and increased propensity of EV-associated α-syn to aggregation potentially due to the presence of gangliosides, have been observed. Such findings implied that α-syn from disease-affected neurons gets transported to healthy unaffected neurons and thereby spreads the disease in a prion-like fashion. Studies have also suggested the role of SUMO in the recruitment of α-syn into EVs and VPS35 in the increased release of EVs containing α-syn into the extracellular space. It is also speculated that EVs released by activated glia containing inflammatory mediators propagate and perpetuate the inflammatory response causing an exacerbation of neuroinflammation contributing to neuronal dysfunction, neurodegeneration, and disease progression. In this context, drugs that inhibit the release of EVs from affected neurons and glia might be beneficial in slowing down the PD progression. A linkage has been observed between the severity of the cognitive impairment in PD with the amount of EVs containing α-syn in the CSF, suggesting that characterization of EVs from the CSF has promise for not only diagnosis but also assessing disease progression in PD. However, due to the invasive procedure of CSF collection, the identification of apt biomarkers in blood-derived EVs would be more appealing as it allows repeated analysis of brain-derived EVs to assess disease progression and treatment efficacy. Indeed, studies have shown higher levels of α-syn in brain-derived EVs in the blood of PD patients, and α-syn levels in such EVs correlated with the disease severity. However, it remains to be determined whether brain-derived EVs in the blood could be employed to detect specific biomarkers related to different stages of PD, particularly Braak stages 1-6 [92]. Although controversial, Braak classification suggests progressive neurodegeneration in brain regions, starting with the olfactory bulb and olfactory nucleus (Stage 1), spreading to the lower brainstem region displaying non-motor symptoms (Stage 2), and substantia nigra pars compacta resulting in typical motor symptoms (Stages 3-4) and ending with the appearance of Lewy bodies in dopaminergic neurons (Stages 5 and 6). Additional markers of interest found in the brain-derived EVs in the blood of PD patients include clusterin, apolipoprotein A1, afamin, apolipoprotein D and J, complement C1r and C1q, IGLV1-33, DJ-1, LRRK2, and multiple miRNAs. However, the variable composition of EVs in different patients has raised questions on their consistency to provide a reliable biomarker. A small number of EVs secreted by disease-specific neurons or glia into the body fluids has also hampered biomarker discovery. Therefore, straightforward, faster, reproducible methods for the extraction, enrichment, purification, and characterization of EVs could help in the accurate and quicker diagnosis and prognosis of PD using brain-derived EVs in the circulating blood.

Regarding the promise of using stem/progenitor cell-derived EVs for treating PD, animal model studies performed so far have implied that MSC-derived EVs could reduce apoptosis, protect dopaminergic neurons, increase dopamine concentration in the striatum and improve motor function. Furthermore, macrophage-derived EVs carrying the antioxidant catalase have been shown to reduce activated microglia in a PD model. Moreover, intravenous administration of RVG-expressing EVs loaded with siRNA against α-syn reduced both α-syn mRNA and protein levels in transgenic mice expressing the phosphomimetic human S129D α-syn, diminished dopaminergic neuron loss, and alleviated PD symptoms. Besides, the administration of dopamine-loaded EVs has been shown to enhance dopamine concentration in the striatum. While the outcome of these studies is advantageous, there are several limitations, which need to be addressed in future studies. The potential mechanisms by which MSC-EVs mediate beneficial effects are unknown, as EVs employed in different studies were not characterized for their miRNA or protein composition. Also, the efficacy of MSC-derived EVs or EVs loaded with catalase, dopamine, or siRNAs was tested only in the early phase of PD in most of these studies. Rigorous studies examining the effects of administration of EVs derived from various stem/progenitor cells or EVs loaded with specific therapeutic compounds or siRNAs with interventions occurring at different stages of the disease are needed to ascertain their efficacy for treating PD.

**Figure 2.**
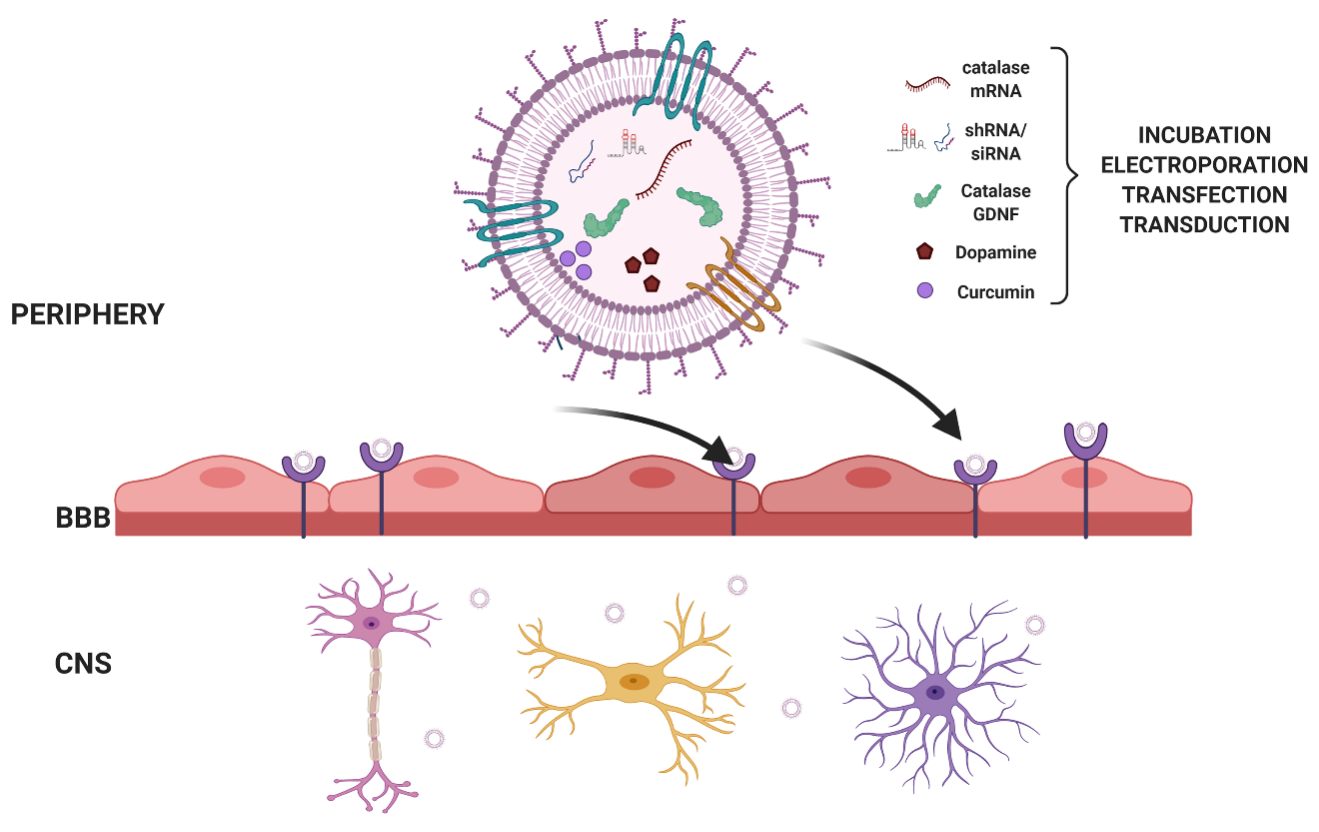
**EVs derived from a variety of cell sources could be loaded with various therapeutic molecules. These include small molecule agonists or drugs, RNA species, and proteins**. Loading of such molecules could be accomplished via simple techniques such as incubation, electroporation, or sonication. Alternatively, endogenous loading could be done by cloning and genetic engineering during EV biogenesis with additional engineering to display protein ligands for the targeted delivery using chimeric proteins consisting of exosomal scaffold proteins such as CD9, CD63 and CD81 fused to the protein of interest. Such engineered EVs could be administered via different routes to deliver the bioactive cargo to neurons and glia to facilitate neuroprotection and modulate neuroinflammation.

EVs derived from a variety of cell sources could be loaded with various therapeutic molecules. Figure 2 illustrates the potential therapeutic agents that could be loaded to EVs for the treatment of PD. These comprise small molecule agonists, RNA species, and proteins. Loading of these agents could be accomplished via simple techniques such as incubation, electroporation, or sonication. Alternatively, loading of molecules could be done through cloning and genetic engineering during EV biogenesis with further engineering to display protein ligands for the targeted delivery using chimeric proteins consisting of exosomal scaffold proteins such as CD9, CD63 and CD81 fused to the protein of interest. Such engineered EVs could be administered via different routes, especially non-invasive, intranasal delivery (IN). However, for a progressive disease like PD that worsens over the years, intermittent administration of drug-loaded EVs into the brain might be beneficial for maintaining sustained clinical benefits. In this context, IN administration appears to be the most promising route because EVs quickly permeate the entire brain after the non-invasive IN treatment (80), and repeated IN administration of therapeutic EVs could potentially be performed in a doctor’s office without anesthesia. Furthermore, advances in the EV field would likely result in the development of nasal sprays containing therapeutic EVs for specific neurological and neurodegenerative diseases in the future, which could deliver the therapeutic cargo of EVs to neurons and glia to facilitate neuroprotection or to modulate neuroinflammation.
